# Supplementary material for: A data flow process for confidential data and its application in a health research project
Source: PLoS One. 2022 Jan 21;17(1):e0262609. doi: 10.1371/journal.pone.0262609 (PMC8782367; doi:10.1371/journal.pone.0262609)
Supplement: S1 Table — (DOCX) [file pone.0262609.s001.docx]

S1 Table. Inclusion Criteria for Patient Selection for the Comprehensive Patient Records project

| **Cohort** | **Cancer** | **Comparator** |
| --- | --- | --- |
| **Inclusion Criteria** | All of the following:   1. Legitimate LTHT relationship 2. A first cancer diagnosis between January 2004 and April 2017 (15 months before extract) at age <= 100 and/or a relevant waiting list flag (gold standard patients; clinically validated cancer cases) (if at age <18, they are deemed a ‘paediatric’ case) 3. Registration (current or historic) with a *ResearchOne* GP or community provider | All of the following:   1. Legitimate LTHT relationship 2. Match the cancer case    1. Sex    2. IMD quintile from current postcode    3. Age: date of birth is within (+/-) 30 months (2.5yr)    4. Paediatric (<18) or adult (>= 18) status on their index date (see below) 3. Have an appointment/event with dermatology, accident and emergency or the *Leeds Dental Institute* within (+/-) 12 months of the matched case’s cancer diagnosis - this is their **index date** 4. Alive 30 days after their index date 5. Alive on the date of the matched case’s first cancer diagnosis 6. Age <= 100 on their index date 7. Registration (current or historic) with a *ResearchOne* GP or community provider |
| **Exclusion criteria** | Any of the following   1. Patient expressed an opt out via *ResearchOne*, *LTHT* or the *NHS Digital* National Opt Out scheme 2. Rare child cancer or rare adult cancer 3. Missing / incorrect NHS number, e.g. no leading zero (for match quality) | Any of the following   1. Patient expressed an opt out via *ResearchOne*, *LTHT* or the *NHS Digital* National Opt Out scheme 2. Cancer diagnosis prior to the matched case’s first cancer diagnosis 3. Missing / incorrect NHS number, e.g. no leading zero (for match quality |
