# Supplementary material for: A data flow process for confidential data and its application in a health research project
Source: PLoS One. 2022 Jan 21;17(1):e0262609. doi: 10.1371/journal.pone.0262609 (PMC8782367; doi:10.1371/journal.pone.0262609)
Supplement: S1 File — (DOCX) [file pone.0262609.s002.docx]

**[Project Name]**

**Data Flow Protocol**

**Table of Contents**

[1. Purpose 2](#_Toc30086202)

[2. Lay Summary 2](#_Toc30086203)

[2.1 OpenPseudonymiser 2](#_Toc30086204)

[2.2 Data Flow 3](#_Toc30086205)

[3. Technical Procedure 4](#_Toc30086206)

[Appendix 1. Worked Example of Pseudonymisation 7](#_Toc30086207)

[Appendix 2. Worked Example of the OpenPseudonymisation Approach 8](#_Toc30086208)

[Appendix 3. Data Providers 9](#_Toc30086209)

[A3.1 [Data Provider 1] 9](#_Toc30086210)

[A3.2 [Data Provider 2] 9](#_Toc30086211)

[Appendix 4. Glossary of Terms and Definitions 9](#_Toc30086212)

# Purpose

This protocol sets out how, for the [project name] study:

- Data will flow from [data type e.g. health records] to a research environment and beyond
- OpenPseudonymiser^[[1]](#footnote-1)^ will be used to enable only authorised linkage

It provides a lay summary and a technical review.

# Lay Summary

The purpose of the [project name] study is to **securely transfer and link [delete if applicable] information that is non-identifiable (not linked to named individuals) from [describe data types e.g. GP records].** This information (known as data) will provide the means to get a very clear picture of [describe study aim]. This protocol explains how data linkage will be done.

The data will be received and linked **[delete if applicable]** from:

- [List the data provider/s, with a short description]

**Appendices 3 and 4** give further information about where the data comes from.

[Data provider name/s] will provide data from [describe the data type and the cohort of individuals the data is about]. The data will be linked at [linkage / ETL organisation]. It will be linked using codes known as digests created using a safe and protected, well-tested process called OpenPseudonymiser. Data will remain securely in the [access site] while it is used for research.

The following sub-sections explain OpenPseudonymiser and how data will be handled during the project.

## 2.1 OpenPseudonymiser

The ‘OpenPseudonymisation process’ enables data linkage without the need for identifiable information. Where datasets previously may have been linked on [identifiable data field/s], OpenPseudonymiser turns the [identifiable data fields] into codes known as digests that can be linked on instead.

OpenPseudonymiser also helps to control what datasets are linked. For example, previously any data with [identifiable data field/s] could be linked in at any point. In contrast, OpenPseudonymiser creates digests for datasets within a project by using a project-specific key. This means that the digest created from an [identifiable data field/s] for one project would not match the digest created from the same [identifiable data field/s] for a different project. As such, the digest could not be used to link data from different projects. This helps to prevent unauthorised or unethical linkage.

## 2.2 Data Flow

Non-identifiable data from [source data type] will be linked and stored in a secure environment and used in research. A computerised process will be used to select the data so that identifiable information is not seen by anyone.

[Data provider name/s] will agree a common ‘key’ (known as a ‘salt’, which is used to generate digests) via direct communication, created using a random digit generator. The salt will only be used for this project. Next, [Data provider name/s] will apply automated procedures to select [study participant data and selection process description] and create a digest for each using the agreed salt and OpenPseudonymiser (Section 2.1).

[Data provider name/s] will securely pass their digests to the [linkage / ETL organisation]. Where the [linkage / ETL organisation] can match the digests, this means that [the / all / both data provider/s have / has] data for these records. [Data provider/s] will produce de-identified datasets from these records and securely deliver encrypted data to the [linkage / ETL organisation].

[delete sentence if linkage is not required:] The [linkage / ETL organisation] will use the matching digests to link the datasets from [Data provider/s]. The [linkage / ETL organisation] will use a different salt to produce a new digest for each linked record via OpenPseudonymiser. This means the digests on the data do not even relate directly to the digests that [Data provider/s] hold. The [linkage / ETL organisation] will also process the data to prepare it for research. They will store the data on [secure server description]. This will yield a single dataset containing all of the data [study participants].

The [linkage / ETL organisation] will grant access to the data for our research team. Access is dependent on signing [linkage / ETL organisation] User Agreement to abide by the ethical and legal requirements of the data. The data remains on [server arrangement] and is accessed and analysed remotely. This means that the data is isolated and a log is kept of who accesses it. Where required data will be securely transferred to secure high performance computing facilities for complex analysis. Where an appropriate data governance framework (e.g. NHS DSPT) is in place data may be transferred ‘as is’. For secure facilities without this, data will be further manipulated with techniques such as replacing disease diagnosis codes with a code only known to the project team to further reduce the risk of re-identification of the data. Data transferred to and from high performance computing facilities will be conducted by the [linkage / ETL organisation] using a secure transfer protocol.

When the research team generate research outcomes and aggregated data that underpins these, they submit these to the [linkage / ETL organisation]. The team check they do not contain identifiable information or in any other way break the ethical and legal requirements of the data. Approved outcomes and data then leave the [linkage / ETL organisation] to inform the public through publications, presentations, public repositories, for example.

# Technical Procedure

The following steps set out the data flow procedure and how OpenPseudonymisation will be utilised. **Figure 1** depicts these steps.

Further explanation is given in **Appendices 1 and 2** to illustrate the OpenPseudonymisation process. **Appendix 3** details where the data comes from. Please refer to **Appendix 4** for a glossary of abbreviations and definitions for OpenPseudonymiser terms such as “salt” and ‘hash’.

1. [Data provider/s] agree a project specific hashed salt (SALT1) created using a “salt” phrase (using a random digit generator).
2. [Describe the data subject selection process]
3. [Data provider/s] use [identifiable data field/s] as identifiers to generate a project specific digest (PSD1) for these records using OpenPseudonymiser and the hashed project-specific salt (SALT1). This process is further explained in Appendices 1 and 2.
4. [Data provider/s] transfer the digests (PSD1) to the [linkage / ETL organisation], where the digest lists are compared and a list of matching digests is compiled (Matched PSD1) [delete if linkage is not applicable].
5. The matched digests (Matched PSD1) are provided to [Data provider/s].
6. The digest lists (matched and original PSD1s) are deleted from the [linkage / ETL organisation].
7. [amend according to the number of providers:] [Data provider 1] produces a de-identified dataset (1D) for the project in an encrypted file and shares it with [linkage / ETL organisation] using [“secure file transfer protocol”].

[Data provider 2] produces a de-identified dataset (2D) for the project. They will encrypt the file and share it with [linkage / ETL organisation] using [“secure file transfer protocol”].

1. [Linkage / ETL organisation] generate a second project specific digest (PSD2) using OpenPseudonymiser and a unique project-specific “salt” (SALT2) to replace PSD1 on 1D and 2D. PSD1 is deleted.
2. [Linkage / ETL organisation] matches the datasets 1D and 2D, generates derived, minimised and aggregated data, placing the research dataset (RD) within a project specific research environment. Data will be screened for re-identification risk.
3. Named members of research team, approved by UoL IRC, are allowed access to the project specific research environment.
4. Research team generate research outputs.
5. [Linkage / ETL organisation] screen for risk of re-identification and approve outputs against the ethical and governance requirements before they leave the [linkage / ETL organisation].
6. Research Team with High Performance Computing needs will be given password protected access to secure facilities for conducting these analyses.
7. The [linkage / ETL organisation] will review all data flowing into and out of High Performance Computing Facilities and conducted transfers via a secure file transfer protocol.


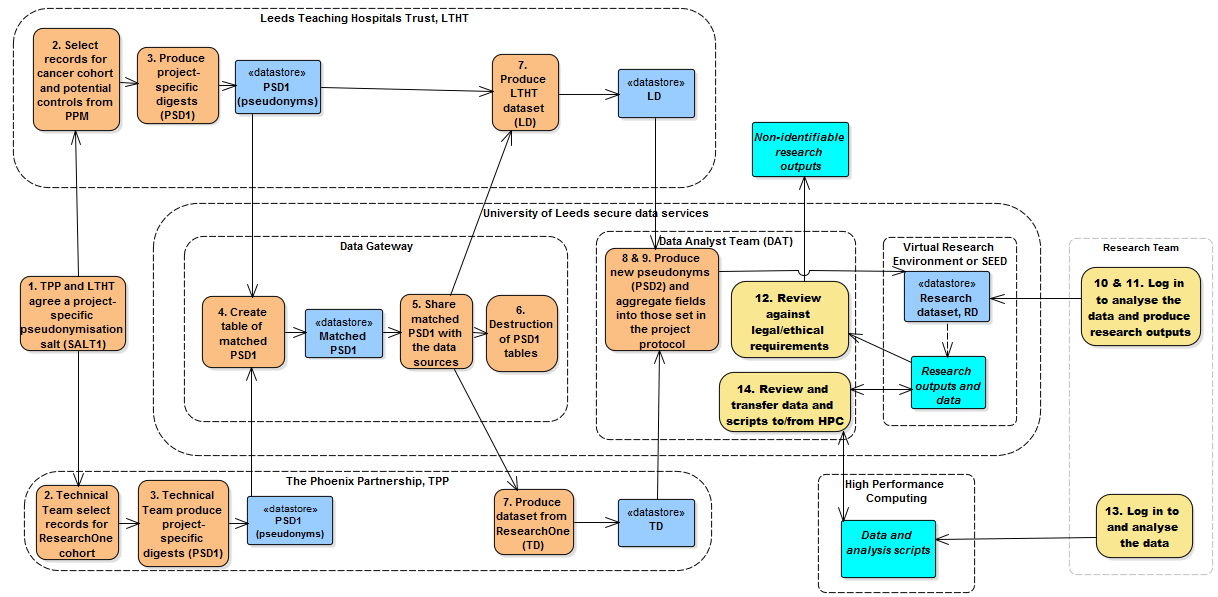
 **Figure 1: Data flow for the project [create a similar that captures the organisations involved]**

**Key**

**Orange, regular:** automated action (no human viewing of data)

**Yellow, Bold:** manual action (human viewing of data)

**Blue <datastore>:** pseudonymous dataset (file with de-identified data in)

***Green, Italic*:** outputs (graphs, papers, algorithms etc.)

--------**:** Secure firewall

**Summarised Glossary** *Further terms are given in Appendix 4*

**Salt**: a random text phrase that is joined to NHS number and month/year of birth prior to OpenPseudonymisation

**Digest**: a ‘pseudonym’ created from the NHS number, month/year of birth and salt during OpenPseudonymisation

**PPM and ResearchOne**: electronic health record databases

**Appendix 1. Worked Example of Pseudonymisation with a Record Identifier [create a similar that captures the identifier/s and organisations involved]**


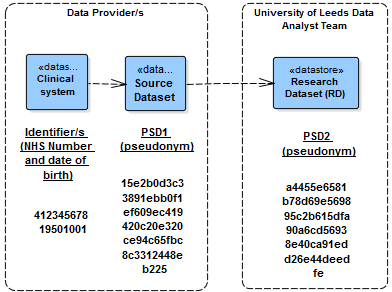


**Key**

--------: Firewall (approval is required to cross this boundary)

This diagram shows how a record identifier (in this case, the fictional [NHS number ‘412345678’ and date of birth ‘19501001’]) would change through pseudonymisation. It shows three stages, which can be described as follows:

- The [data provider/s] have [data source/s] that hold the identifiers ([identifier type]).
- This is replaced by a digest (PSD1 *in Figure 1*) in the source-produced datasets ([xxx e.g. LD and RD] *in Figure 1*).
- UoL IRC then replace PSD1 with a new digest (PSD2 *in Figure 1*) in the research dataset (RD *in Figure 1*).

# Appendix 2. Worked Example of the OpenPseudonymisation Approach


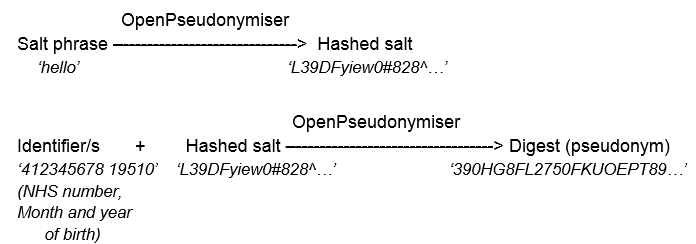


This diagram shows how a project-specific digest (pseudonym) is produced, with a worked example. Please refer to Appendix 4 for a glossary of terms and definitions.

**[Data provider/s]** will use this process to produce PSD1 (*in Section 3*) from identifiers plus SALT1 (*in Section 3*), as follows:

- [Data provider/s] will download OpenPseudonymiser / source code from the University of Nottingham website <https://www.openpseudonymiser.org/>.
- [Data provider/s] will agree on a project-specific SHA-256 hashed “salt” (SALT1), created using OpenPseudonymiser.
- This salt is effectively ‘extra data’ and the data sources use this plus an agreed identifier such as NHS number and OpenPseudonymiser to produce project-specific digests that act as pseudonyms (PSD1).
- [Data provider/s] will only use SALT1 to produce datasets for this project. They will not share SALT1 further without explicit ethical approval.

[Linkage / ETL organisation] will use this process to produce PSD2 (*in Section 3*) from PSD1 plus SALT2 (*in Section 3*), as follows:

- [Linkage / ETL organisation] will download OpenPseudonymiser / source code from the University of Nottingham website <https://www.openpseudonymiser.org/>.
- [Linkage / ETL organisation] will produce a project-specific “salt” phrase with which they will use OpenPseudonymiser to create a SHA-256 hashed “salt” (SALT2).
- LTHT and TPP provide [linkage / ETL organisation] with project datasets (1D and 2D) that contain PSD1.
- [Linkage / ETL organisation] will produce specific digests (PSD2) for the research dataset (RD), using SALT2, PSD1 and OpenPseudonymiser.
- [Linkage / ETL organisation] will only use SALT2 to produce research datasets for this project. They will not share SALT2 further without explicit ethical approval.

# Appendix 3. Data Providers

The data required for this project is contained within [provide summary describing the data types and what will be gained from linkage / transfer].

## A3.1 [Data Provider 1]

[Summarise the organisation, its data and research dataset preparation process. Define the ethical framework for data extraction and use in this research]

## A3.2 [Data Provider 2]

[Summarise the organisation, its data and research dataset preparation process. Define the ethical framework for data extraction and use in this research]

**Appendix 4. Glossary of Terms and Definitions**

Below is a **list of abbreviations** used in this protocol and their full terms:

| **Abbreviation** | **Full Term** |
| --- | --- |
| [Fill in for any abbreviations used in describing the data provider/s and data linkage / ETL organisations] | |
| SALT1 | Project specific “salt” used by LTHT and TPP to produce project specific digests |
| PSD1 | Project specific digests produced by LTHT and TPP to enable pseudonymous linkage |
| 1D | A project-specific de-identified dataset from [data provider 1] |
| 2D | A project-specific de-identified dataset from [data provider 2] |
| SFTP | “Secure file transfer protocol” is a network protocol for file transfer over a secure data connection |
| SALT2 | Project specific “salt” used by [linkage / ETL organisation] to produce project specific digests |
| PSD2 | Project specific digests produced by [linkage / ETL organisation] to provide the research team with patient-level digests in the research dataset (for the purpose of patient-level analysis) that do not link directly back to digests held by [data provider/s] (PSD1) |
| RD | The research dataset viewed by the research team within the [data handling organisation] |

Below is a **list of terms** and their definitions as used herein. These have been informed by the following documents:

- DD ISO/TS 25237:2008
- HSCIC Code of Practice on Confidential Information
- ICO Anonymisation Code of Practice

| **Term** | **Definition** |
| --- | --- |
| Identifiable data | Data that may reasonably be expected to include information that may identify a living or deceased individual, or may relate to an individual when combined with other fields either within the dataset or that may reasonably come into the possession of the approved data recipient. |
| Non-identifiable (or de-identified) data | Data in a field or dataset that does not enable an individual to be identified using reasonable effort. It may be created from identifiable data through the process of anonymisation or pseudonymisation. |
| Pseudonymisation | Processing that is applied to agreed fields within datasets to enable linkage between agreed datasets by means of a pseudonymous digest. |
| Digest | A digest is a ‘pseudonym’ created from agreed data fields during pseudonymisation. |
| Salt | A salt is a random text phrase that is joined to field(s) (such as NHS number) prior to pseudonymisation. |
| Hash | A cryptographic function used to change data in a way so that it is practically impossible to re-convert back. |
| SHA-256 | Secure Hash Algorithm 256 is an industry-standard hash function. |
| Project specific salt | By adding a salt that is unique to each project to the fields being pseudonymised, the resultant digests differ for each project. This helps to prevent data being linked across project boundaries. |
| Project-specific digest | When a digest is created using a project-specific salt, it enables linkage between agreed datasets for an agreed purpose. Datasets with digests produced using a different salt would not link across. |
| Patient-level digest | This is a pseudonymous digest that is patient-specific. This enables a) the recipient to interpret the data at the patient level and b) the data provider to re-identify the patient for legal, health or safety purposes. |
| Anonymous data | Non-identifiable data that is not linkable to other datasets. Anonymous data should be handled in conditions that are appropriate to maintain its anonymity. |
| Pseudonymous dataset | A non-identifiable dataset that contains otherwise anonymous data linked to a pseudonymous digest. It can only be linked to datasets containing digests produced in the same way. Such data should be accessed and stored in conditions that are appropriate to maintain its non-identifiability. |
| OpenPseudonymiser | Pseudonymisation software used to produce project-specific encrypted salts and digests. It is a University of Nottingham application that utilises SHA-256 (Secure Hash Algorithm). |

1. https://www.openpseudonymiser.org/ [↑](#footnote-ref-1)
