# Supplementary material for: A data flow process for confidential data and its application in a health research project
Source: PLoS One. 2022 Jan 21;17(1):e0262609. doi: 10.1371/journal.pone.0262609 (PMC8782367; doi:10.1371/journal.pone.0262609)
Supplement: Attachment — Submitted filename: Response to Reviewers.docx [file pone.0262609.s003.docx]

Response to Reviewers

**Thank you for the feedback and opportunity to address the comments raised. We have added our response to all points raised by the reviewers in line below, in bold font. Line references correspond to the attached ‘Revised Manuscript with Track Changes’.**

Reviewer #1: Linking the research datasets of patients without divulging the sensitive medical information of patients and hospitals is very difficult. This research work, in this line, has proposed a dataflow protocol for anonymous linked datasets for research purposes. It is accomplished by Comprehensive Patient Records study in Leeds, United Kingdom with data from two providers which contains the details of 365, 193 patients.

The protocol design has been verified by research advisory groups.

The authors have received the legal advice for this protocol that it maintains ethical as well as legal requirements.

This protocol will be very useful during the Covid-19 situations to analyze and identify any useful patterns.

The use of OpenPseudonymiser for creating secure digests and linking the digests while preventing backward engineering seems to be very useful.

**Many thanks for your review and feedback.**

Reviewer #2: Authors presented a technological impact of trust in Social Government during COVID-19 crisis. Though, paper covers important topic, technical contribution of this paper is limited. Also, following comments need to be addressed in the revised manuscript:

-Highlight your contribution clearly in Abstract section.

**We believe that this manuscript contributes a linkage and anonymisation approach with clear steps for creating a research dataset in line with ethico-legal requirements. We also produced a research dataset that has important value for cancer research and describe how it has been made accessible.**

**We have now clarified this by highlighting the issue, study aim and contribution in the abstract (lines 28-30, 34-36 and 52-53).**

-Mention the quantitative findings of this research in the abstract section.

**The main findings of the research were that a) a data flow protocol was defined for linking and anonymising data with ethico-legal approval; and b) the data flow protocol was successfully applied in a case study in which data regarding 365,193 patients was linked and anonymised from two healthcare providers. The invaluable cancer dataset that was created is now available for use in research**

**We have now highlighted this more clearly in the abstract (lines 43-49)**

-Fig. 1 is not explained properly.

**Thank you for the opportunity to provide further clarity. The data flow protocol that is defined in the manuscript was utilised in a case study. Figure 1 depicts the parties involved in data handling in this case study: the data transfers, the linkage and anonymisation actions performed, and the research access granted to the final (linked and anonymised) research dataset.**

**We have now added a description of the information in Figure 1 (lines 289-290) and further clarified the caption for Fig 1 (lines 308-309).**

-Also, in some figures, captions are not proper.

**We have now amended the captions for both Fig 1 and Fig 2, by adding further descriptive detail (lines 307-312).**

-Introduction section needs to be re-written to improve its quality and readability.

**The Introduction section has been refined throughout, to clarify the issues that the manuscript addresses and to improve readability (lines 65-67, 76-80, 86-87, 93-99, 106-114, 119-123, 132-136).**

-Put some more light on concept of the paper and its usability to the industry and society.

**As described above, we have now defined the aim and contribution more clearly in the Abstract, and also in the Conclusions section as described in response to a further comment below. We have also elaborated on the concept of the paper and its contribution to industry and society in the Introduction (lines 132-136) and Discussion (lines 316-322).**

-Paper needs to polish and provide a detailed explication of theoretical aspects such as conditions and theorems, and practical issues like algorithms, rules and possible applications.

**We have now refined the flow of this paper through general changes throughout. We have also now explicitly referenced the technical detail of the data flow and worked examples of pseudonymisation that are provided in the Supporting Information (lines 279-281). The glossary of terms in the Supporting Information, which provides further explication, is also now referenced. Further, we have now highlighted the possible application of the described approach in addressing practical issues in enabling the secondary use of data for research (e.g. Abstract, Conclusions, and lines 316-322). We have made explicit that the protocol is shared and access to the CPR dataset is made available, both in order to assist in future research projects (lines 268, 321-324).**

-Following are some of relevant and recent references which can be referred in the revised manuscript:

Provably Secure Data Sharing Approach for Personal Health Records in Cloud Storage Using Session Password, Data Access Key, and Circular Interpolation,

Capability based outsourced data access control with assured file deletion and efficient revocation with trust factor in cloud computing,

IoT-based big data secure management in the fog over a 6G wireless network,

Efficient escrow-free CP-ABE with constant size ciphertext and secret key for big data storage in cloud,

A novel CNN based security guaranteed image watermarking generation scenario for smart city applications.

**Thank you for providing references to these manuscripts, which have now been cited as relevant (lines 74-75).**

-In the conclusion section, I suggest, to highlighting the new discovery, new inventions and new aspects of the research.

**The manuscript contributes a) a data flow protocol for linkage and anonymisation, b) description of a case study that successfully utilised the data flow protocol, in the healthcare domain, and information on how to request access to the resultant dataset; and c) a proposed approach and recommended criteria for cross-accreditation between organisations that handle data.**

**The Conclusions section has now been amended to highlight these three contributions (lines 472-481.**

-Improve overall flow of this paper for better understanding.

**We have now improved the flow of this paper to aid understanding, through general changes applied throughout the manuscript.**
