# Supplementary material for: A data flow process for confidential data and its application in a health research project
Source: PLoS One. 2022 Jan 21;17(1):e0262609. doi: 10.1371/journal.pone.0262609 (PMC8782367; doi:10.1371/journal.pone.0262609)
Supplement: Attachment — Submitted filename: Response to Reviewers.docx [file pone.0262609.s004.docx]

Response to Reviewers

**Thank you for the feedback and opportunity to address the comments raised. We have added our response to all points raised by the reviewers in line below, in bold font. Line references correspond to the attached ‘Revised Manuscript with Track Changes’.**

Reviewer #1: The proposed work is about the utilization of valuable health data of patients. The idea of patient confidentiality is clearly maintained by anonymizing the data and linking them together for research and other application purposes. The authors claim that, the work has potential applications not only in England and European Union as the handled datasets is pertaining to these geographical locations, but to the global scale.

But, I suggest the authors clarify in what way is this research work related to security.

**As described in lines 432-435, use of the protocol encourages consideration of the wider information security and governance framework, including the adoption of mechanisms for secure data transfer and restricting data access as appropriate. We acknowledge that the research has not directly developed any security feature, but rather the research work relates to security through guiding protocol users through the steps and security measures to be considered during linkage and anonymisation exercises.**

**We have therefore now clarified that the protocol relates to the linkage and anonymisation of data, rather than security directly (lines 299, 305).**

Apart from this, many worthwhile contributions such as

1. "An efficient anonymous authentication and confidentiality preservation schemes for secure communications in wireless body area networks"

2. "A new SmartSMS protocol for secure SMS communication in m-health environment"

3. "An efficient anonymous authentication and key agreement scheme with privacy-preserving for smart cities"

to be added to the review section of this article to make it oriented towards confidentiality and security concerns.

**Thank you for these references. We have now discussed and cited these as appropriate in the manuscript (lines 73, 112-113, 406-408, 423-424).**

As a whole, this research work is a novel proposal by the authors and if the suggestions are incorporated, the paper can be considered for possible publication in your esteemed journal.

**Many thanks for your review and guidance.**

Reviewer #2: A data flow process for confidential data and its application in a health research project

is presented in this paper. Paper is revised well. It can be accepted now.

**Many thanks for your review and feedback.**
